# Capturing Behavior in Small Doses: A Review of Comparative Research in Evaluating Thin Slices for Behavioral Measurement

**DOI:** 10.3389/fpsyg.2021.667326

**Published:** 2021-04-29

**Authors:** Nora A. Murphy, Judith A. Hall

**Affiliations:** ^1^Department of Psychology, Loyola Marymount University, Los Angeles, CA, United States; ^2^Department of Psychology, Northeastern University, Boston, MA, United States

**Keywords:** thin slice, predictive validity, behavioral coding, nonverbal behavior analysis and synthesis, reliability

## Abstract

Thin slices are used across a wide array of research domains to observe, measure, and predict human behavior. This article reviews the thin-slice method as a measurement technique and summarizes current comparative thin-slice research regarding the reliability and validity of thin slices to represent behavior or social constructs. We outline decision factors in using thin-slice behavioral coding and detail three avenues of thin-slice comparative research: (1) assessing whether thin slices can adequately approximate the total of the recorded behavior or be interchangeable with each other (representativeness); (2) assessing how well thin slices can predict variables that are different from the behavior measured in the slice (predictive validity), and (3) assessing how interpersonal judgment accuracy can depend on the length of the slice (accuracy-length validity). The aim of the review is to provide information researchers may use when designing and evaluating thin-slice behavioral measurement.

## Introduction

Observing and measuring behavior is foundational to behavioral research (Greene, 1941; Vaughan, 1948). The measurement of behavior to understand features of communication and person perception is widespread across many domains such as psychology, sociology, medicine, and communication. In this article, we review the thin-slice method as a behavioral measurement technique and review comparative thin-slice research (Ambady et al., 2000; Slepian et al., 2014; Murphy et al., 2015).

Thin-slice methodology refers to utilizing a small excerpt from a longer behavioral stream. This means, for the researcher, either deciding at the outset to record or gather very limited amounts of behavior (for example, recording only the 1st min of an interaction even though the interaction is much longer), or making a later decision to analyze, or present to viewers, only short excerpts from all the recorded or transcribed material that one possesses. Typically, an interaction is video or audio recorded and then slices are extracted from those recordings or their respective transcripts. The interaction or “behavioral stream” can be of any length, and while there is no fixed definition of what constitutes a “thin slice,” thin slices typically are under 5 min. The thin-slice excerpt then can be coded or rated for behaviors or characteristics of individuals (targets) in the interaction. Thin slices also may be shown to viewers who judge a target's state or trait, if the goal is to assess judgment accuracy. The idea is that the slice is representative of a target's behavior throughout the interaction and/or that the slice may reveal or predict a target's internal states, personality, or other social attributes. In this article, we review comparative thin-slice research involving dynamic stimuli^1^, which typically involves comparisons about different slice lengths (Murphy, 2005; Murphy et al., 2015; Krzyzaniak et al., 2019), as well as examination of slice locations (Carney et al., 2007; Fowler et al., 2009; Wang et al., 2020).

Thin slices are used to code target behavior (i.e., how is the person behaving) or as stimuli in person perception research, wherein observers make inferences about targets based on their behavior. Behavioral researchers are usually drawn to thin-slice techniques out of sheer pragmatism—to ease coding burdens, reduce viewer time, and in general to make the best use of limited resources of time and patience among research personnel and research participants (Murphy, 2005, 2018). The practical benefits of the thin-slice method are clear. Given the inherent complexity of behavior, thin-slice methods ease the burden of behavioral measurement because measuring behavior is an arduous task. Various researchers' descriptions of dynamic behavioral coding include: “time-consuming,” “labor-intensive,” “tedious,” “costly,” “complex,” “challenging,” “painstaking,” “mentally-straining,” “inefficient,” “serious commitment,” and “daunting,” among many other unfavorable terms (Gosling et al., 1998; Murphy, 2005; Black et al., 2013; Fujiwara and Daibo, 2014; Carcone et al., 2015). One way researchers deal with the time-consuming nature of behavioral coding is to ask coders or raters to watch or listen for several behaviors at the same time—for example, to simultaneously count smiles and head tilts, or to simultaneously rate anger, anxiety, and sadness (e.g., Wang et al., 2020). This may not be optimal because it divides the observer's attention and may encourage inflated correlations among the behaviors or attributes being coded or rated.

Choosing not to employ thin slices could exponentially increase coding or rating time, depending on what is being measured and the length of original recordings (Murphy, 2005). Some coding projects are impressively colossal in scale; for example, Bensing et al. (2008) employed two coders both of whom timed gaze by physicians toward their patients for the entirety of some 2,000 patient visits that averaged about 10 min each. Fairbairn et al. (2013) coded 7.9 million frames of video data from 92 participants engaged in 36-min interactions. For obvious reasons, therefore, researchers actively seek techniques to reduce the burdens of coding. Simply put, thin-slice behavioral measurement is easier than coding a longer behavioral stream.

Automated coding using software or equipment is another approach to reducing coding labor (Georgiou et al., 2011), because such systems can sometimes eliminate the human element and are unlikely to be limited by the duration of the stimuli. Existing technology can automatically extract nonverbal features such as prosody, turn-taking, pauses, gesturing, interactional synchrony, and nodding (Fujiwara and Daibo, 2014; Nguyen and Gatica-Perez, 2015; Lausberg and Sloetjes, 2016; Ramseyer, 2020). Machine-learning methods can train a computer model to recognize behavioral features (e.g., a smiling face) based on a small corpus of recorded behavior (Chakravarthula et al., 2021). Such automated approaches can considerably reduce manual coding time, though many still require human coders (Narayanan and Georgiou, 2013; Girard et al., 2015).

While automated methods are attractive, they are not a panacea for reducing coding burdens (Schmid Mast et al., 2015). These sophisticated methods rely on an interdisciplinary approach often involving computer scientists, statisticians, and behavioral researchers. Learning how to implement new software or equipment, which is often expensive, requires a steep learning curve. As these techniques advance, the time and training needed to learn such automatic approaches will likely decrease. For now, there is a trade-off between learning and paying for automated methods and the time to complete traditional manual coding. Another potential limitation of automated methods is the inability to code molar constructs that involve the extraction of meaning from an integration of behavioral cues (e.g., friendliness, anxiety, or competence). In fact, it can be an error to assume that exact measurement such as provided by automatic methods equates to *psychologically meaningful* measurement; behavioral researchers often want to know what movements mean, not just how often they happen or what they look like (Funder and Colvin, 1991; Blanch-Hartigan et al., 2018).

Thus, researchers may turn to thin slices as a desirable coding technique and the use of thin slices across many domains is a testament to the method's versatility. Yet, inherent to the thin-slice technique are questions about the reliability and validity of thin slices to represent behavior or social constructs such as: How well do thin slices capture the whole of a behavioral stream? Are they interchangeable with each other? How well do thin slice measurements correlate with different (external) variables, compared with the totality of the recorded behavior? How much does accuracy of judging targets' states and traits depend on the duration of the stimuli shown to perceivers?

While our present goal is to provide potentially useful information to academic researchers, there are real-world applications where more knowledge about thin slices could be important. As examples, Perrault (2020) compared slices of different lengths from introduction videos made by physicians for potential patients, in order to determine how long such videos should be in terms of viewers' responses and attention span. Hall et al. (2009, 2014), in studies of medical visits and corporate technical support calls respectively, found evidence for the importance of the very first min or two of the interaction in predicting important patient or client outcomes. In fact many studies using thin slices have been conducted in clinical psychology, medicine, and business, demonstrating that thin-slice research could have meaningful impact in domains far removed from the psychology lab.

Truth be told, however, there is little rhyme or reason to the many choices researchers make about thin-slice coding, such as deciding an appropriate slice length and from where in the interaction should the slice be extracted. Using thin slices rests on an assumption that the methodology itself is a reliable and valid representation of behavior, but making these decisions is often an act of faith on the part of researchers. As with any measurement tool, empirical conclusions are only as strong as the reliability and validity of such methods (Flake et al., 2017). This article will review thin slices for behavioral measurement, describing thin-slice coding techniques and reviewing comparative research of several kinds. We cannot describe in detail the many studies that have employed thin slices, as there are far too many. Rather, we will focus on research that is aimed at understanding the *trade-offs* involved in longer vs. shorter slices, or slices from different temporal positions within the behavioral stream, so that researchers can exercise more rationality (i.e., go beyond pragmatism or guesswork) when designing and evaluating research. Efforts to establish reliability and validity of thin slices by comparing results obtained for slices of different lengths—what we call *comparative thin-slice research*—is a relatively new undertaking^2^. We will focus on three tacks in which comparative thin-slice research has been done: (1) assessing whether thin slices can adequately approximate the total of the recorded behavior or be interchangeable with each other (which we call *representativeness*), (2) assessing how well thin slices can predict variables that are different from the behavior measured in the slice (which we call *predictive validity*), and (3) assessing how interpersonal judgment accuracy can depend on the length of the slice (which we call *accuracy-length validity)*.

## Historical Roots and Modern Uses of Thin-Slice Methods

The term “thin slice” was coined by Ambady and Rosenthal (1992) in a meta-analysis on correlations between thin-slice coding or rating and outcomes of interest, such as depression, ratings of teacher effectiveness, or medical patients' satisfaction^3^. That article (cited more than 2,200 times as of January, 2021)^4^ demonstrated thin slices to be a method that is widely and justifiably used, and established the thin-slice methodology as a topic of research in its own right.

Of course, thin slices had been in use for many years before they received their name. For example, Waxer (1974) used 2-min silent video clips of individuals in psychiatric interviews to find out whether naïve viewers could recognize those with depression. Milmoe et al. (1967) used ratings of electronically filtered audio clips of under 2 min (and their respective transcripts) to predict therapists' success in referring alcoholics for treatment. Ekman et al. (1980) used both 1 and 2-min video slices for judgments of honest and dishonest interviewees. Hall and Braunwald (1981) obtained impression ratings of male and female speakers based on 10-s audio clips from television shows to find out if listeners could tell whether a male or female was being spoken to and what vocal qualities the speakers used.

To observe and measure behavior, researchers code and analyze interactions of all sorts, such as interactions between parent and child, relationship partners, or strangers in get-acquainted sessions. Coding schemes are often employed and typically capture many constructs but the schemes often involve lengthy training periods in addition to the actual coding time itself. For instance, the Living in Familial Environments coding system (LIFE) involves more than 40 separate coding units, such as caring and irritated, with one study describing a training protocol as lasting 6 months (Hops et al., 1987). As another example, the Motivational Interviewing Skills Code scheme (MISC; Miller and Rollnick, 2002) was used to assess therapist and client functioning and one study reported that training coders took more than 40 hr across 4 weeks (Moyers et al., 2003).

Motivated by easing coding burdens, some researchers only record relatively short episodes of behavior, instead of analyzing slices of longer recordings. At a technical level, such approaches do not involve thin slices as slices are not being extracted from a longer interaction. Yet, these measures do align with theoretical perspectives that short interactions (5 min or less) can reliably capture behavior and other social constructs. As one example, communication constructs such as cognitive sensitivity and responsivity were reliably captured from 5-min parent, child, and/or sibling interactions (Prime et al., 2014, 2015; Sokolovic et al., 2021). The research design was developed specifically to provide quick, cost-effective, and validated measures of communication styles and the authors concluded that using a thin-slice approach (i.e., using interactions <5 min) is a viable alternative to coding longer interactions. Yet, as valuable as such approaches may be, they do not answer the question of whether 5 min is better than, say, 1 or 2 min, or worse than 10 or 15 min^5^.

## Theoretical Perspectives on Behavioral Expression Within Thin Slices

Beyond the practicality of thin slices, there is confidence to be gained in the legitimacy of thin slices to represent behavior as thin slices are related to a number of larger theories about behavior in social interactions. There is an evolutionary advantage to drawing inferences about a person from glimpses of their behavior. Just as primate expressions involving shrieks that signal anger and potential attack (Chevalier-Skolnikoff, 1973), a loud voice is perceived as and validly indicates social dominance in humans (Hall et al., 2005). These brief behavioral expressions (in essence, thin slices of behavior) provide information for a perceiver to act upon, potentially conferring an evolutionary advantage of making decisions about approach or avoidance, communication, and further interaction (Zebrowitz and Collins, 1997). Being able to make judgments and decisions based on little information is required for functional daily life. For instance, racial bias is detectable via thin slices (Richeson and Shelton, 2005) and knowing such information is undoubtedly of value in making interaction decisions.

Theoretical support for thin slices is also evident in the bedrock of personality science, behavioral consistency, whereby an individual's behavior is consistent across situations and time. That is, traits will exist within an individual with some regularity across various situations, such as at work and at home (Epstein, 1979). Underlying this tenet is the idea that personality is evident in behavioral expressions, and a host of research supports this notion (Allport, 1937; Murphy, 2007; Leikas et al., 2012; Letzring et al., 2021). While acknowledging situational variance, research persistently supports behavioral consistency in the expression of personality (Funder and Colvin, 1991; Shoda, 1999; Fleeson and Law, 2015; Geukes et al., 2017). Thus, the use of thin slices, as small glimpses into a person's behavior, fits within the behavioral consistency premise in personality science.

While not quite a theory, Egon Brunswik applied his existing visual perception paradigm to social situations, and the Brunswik lens model is often applied in understanding social perception processes (Brunswik, 1956; Hall et al., 2019). The Brunswik lens model specifies that individual behavioral cues are related to impressions of a target as well as a target's actual personality or other characteristic of interest. Behavioral cues that relate to observer impressions of a target provide insight into observers' implicit theories about the trait or characteristic in question. Likewise, cues related to a target's measured state or trait provide insight into how states or traits are revealed through behavior. Measurements of behavior are an essential feature of the Brunswik lens model and thin slices provide that opportunity. In sum, beyond the practicality of using thin slices for behavioral coding, a number of larger theories of human interaction support the notion that thin slices are appropriate reflections of human behavior and, in turn, offer support to a researcher's decision to adopt thin slices as a behavioral measurement tool.

## Deciding to Use Thin Slices for Behavioral Measurement

As with any decision to use behavioral coding, a number of considerations should be taken into account deciding whether thin-slice coding and stimuli are appropriate to a research question or design (e.g., Baucom et al., 2017; Blanch-Hartigan et al., 2018). Here, we outline some topics a researcher might consider in making such decisions. At a basic level, any coded behavior or construct needs to be *observable* (Funder, 1995; Ambady et al., 2000). Is there existing evidence that the construct of interest is (potentially) observable at all? For instance, while extraversion may be easily judged from thin slices, agreeableness is harder to detect, and thus, potentially harder to code from thin slices (Ames and Bianchi, 2008). Likewise, the observability of a behavior or construct within the specific setting of the behavioral stream should be considered. Is it reasonable to expect that attraction or suggestibility would be evident in a medical setting or interactions with children? For instance, Whalen et al. (2020) found that obtaining thin-slice reliability for judging preschoolers' personality traits varied between structured tasks (e.g., unwrapping an empty box compared to telling a story). The authors noted that certain tasks may have restricted the range of expression, in turn making personality harder to observe and reliably measure from thin slices in those particular settings. Thus, a researcher should think about the potential observability of a behavior or construct of interest within the specific research setting.

When researchers design lens model studies, they must choose what behaviors to code and include in their models. There can be wide variation in the “success” of such a model, depending on whether the cues have a priori likelihood of being related to the criterion (trait or state) and/or to judges' impressions. When extensive prior research enables the researcher to pick highly diagnostic cues, they will likely find a great deal of evidence for how accurate judgments are mediated by specific cues (e.g., Laukka et al., 2013). In contrast, a study that is more exploratory might find there is accurate judgment but fail to identify any validly utilized cues because, presumably, judges were able to validly utilize cues that the researcher did not measure (e.g., Ruben and Hall, 2016). Though these examples do not speak to the intrinsic wisdom of using thin slices, whether the lens model has high explanatory strength is a matter of the coded cues' theoretical and empirical relevance to the trait or state being studied.

Researchers also should also consider the *consistency* and *frequency* of behavioral expressions across and within interactions and across settings. Some nonverbal behaviors seem to be expressed more consistently across interactions as compared to others. For instance, gaze and nods showed relatively consistent expression within interactions and across various interaction settings (e.g., zero-acquaintanceship dyadic interactions, job interviews, medical settings) (Patterson, 1973; Leikas et al., 2012; Murphy et al., 2015). However, more variability has been found for behaviors such as speaking time, indicating that (shorter) slices may not adequately capture or be representative of that behavior across an interaction or setting. Relatedly, the possible frequency of a behavior is pertinent. A behavior such as crossed arms may occur less frequently as a whole and thus capturing that behavior and its representativeness within slices may be less likely.

Another important decision is whether to code *molar constructs* and/or *micro behaviors*. Molar constructs refer to higher levels of abstraction and may be more holistic in nature. Examples of molar constructs include dominance, awkwardness, perceived intelligence, or pleasant style of speech. Often, such constructs would use Likert-style ratings for measurement. On the other hand, individual (micro) behaviors that are coded descriptively, not requiring much if any coder inference, may be more concrete or exact, as they usually represent specific behaviors or expressions, such as number of smiles, duration of eye gazing, or speaking time. While individual behaviors such as smiling or gazing also can be measured with Likert-style ratings (e.g., Briton and Hall, 1995; Wang et al., 2020), often such behaviors are measured as frequency counts or duration. The decision between coding molar constructs or micro behaviors depends entirely on what the researcher's interests are. Micro behaviors may suit some research goals whereas molar judgments (e.g., ratings of awkwardness, sincerity, or truthfulness) may better answer other research questions (Funder and Colvin, 1991; Leikas et al., 2012). Conceivably, optimal slice lengths might vary depending on how much inference coders or raters are required to make. One overarching aspect in aforementioned thin-slice coding decisions is whether topics of interest are affective states or traits. States are more temporary experiences while traits are more stable across time and situations (Augustine and Larsen, 2015). The questions of observability, frequency, duration, molar constructs, and micro behaviors may all tie into whether the construct of interest is a state or trait, though a wide array of both states and trait research has employed thin-slice methods, suggesting that thin slices may be appropriate to either.

## Comparative Thin-Slice Research

Three strands of comparative thin-slice research will be described in the following sections: *representativeness, thin-slice predictive validity*, and *accuracy-length validity*. It must be said at the outset that in all three of these domains there is much methodological variation across studies—in the behaviors that are measured and the constructs that are judged, the outcome variables used for prediction, specific measurement methods (e.g., molar vs. micro), slice lengths, temporal position of the slice, length of the total recorded behavior, and other variables, meaning that no one study can settle questions regarding optimal slice lengths or slice locations. All we can provide is an overview in the hope that some knowledge on these questions is better than no knowledge at all.

Also important is that, for most purposes, the relevant metric for interpretation is the magnitude of the correlations being compared, not whether they are statistically significant, as the latter is tied to the irrelevant (for these purposes) factor of sample size. The challenge for a researcher who is interpreting such correlations, or planning a study, is to decide whether the relevant correlations are big enough, or similar enough, according to their own criteria to justify using thin slices rather than the “total” behavior, whatever that may be. An illustration regarding predictive validity will help. Let us say that total interpersonal gaze in a 5-min interaction predicts observers' ratings of likeableness at *r* = 0.30, while the same correlation based on a 1-min slice of that 5-min interaction is *r* = 0.26. There is some loss in magnitude of prediction, to which the researcher would apply their own decision rule. One researcher might decide the loss is too much and opt to stick with the “total” gazing measurement, or perhaps decide to use a 2-min slice that yielded a predictive correlation of *r* = 0.29. Another researcher might decide that the loss of magnitude by using the 1-min slice is well worth the savings in personnel time and cost. Of course, the researcher can also conduct a power analysis to decide what the sample size should be if they want to conduct inferential statistical tests (Abraham and Russell, 2008). Ultimately, it is up to an individual researcher to decide which approach fits their needs.

Finally, for studies looking at representativeness and predictive validity, it goes without saying that a great deal depends on the psychometric quality of the behavioral coding (e.g., Moskowitz and Schwarz, 1982). Slices with strong intercoder reliability will show more promising evidence for the value of the slices than slices with weak intercoder reliability—so a general statement of “slices work well” or “slices don't work well” could easily be confounded by the psychometric quality of the slices, not the slice length or location *per se*. Poorly measured slices will not correlate well with each other or with other variables. Increasing the number of raters is an easy way to improve reliability, yet leads us back to the issue of labor and time. Other sources can provide more detail and guidance in assessing and improving psychometric quality (for example, by employing more and/or better trained coders) (Li et al., 1996; Rosenthal, 2005).

### Representativeness

Representativeness refers to the ability of one slice to be interchangeable with another slice, or to adequately represent the “total” behavior. Both of these aspects of representativeness are addressed via correlations, often using the same statistics that researchers commonly used for assessing reliability (e.g., coefficient alpha, intraclass correlation, corrected part-whole correlations). For that reason, sometimes representativeness is referred to as reliability but sometimes it is referred to as validity when the question is the correlation between a slice and the total (because the total is operationally defined as the ground “truth” and the correlation of the slice to the total indicates the validity of the slice) (Murphy et al., 2015).

It is important to note that the reliability discussed here is different from the reliability of coders (as discussed in the previous section) or the internal consistency of items in a coding scheme. Also, readers should be clear that we are not concerned with comparisons of mean levels of any given coded or rated behavior (for example, whether the amount of smiling varies across slices). While that question is of great interest for some research purposes (e.g., Ruben et al., 2015), it does not speak to the representativeness of slices, which is assessed via inter-slice correlations or by slice-total correlations.

#### Inter-Slice Reliability (Interchangeability)

Here the question is whether the individuals whose behavior is measured maintain their rank in the distribution from slice to slice—for example, if Jacinda smiles a lot (relative to other people) in the first slice, does Jacinda also smile a lot (again, relative to others) in other slices? If this kind of reliability is high, it means that people's relative amount of the behavior is well captured in any slice.

Hall et al. (2009) extracted three 1-min slices from early, middle, and late in a 15-min medical interview and obtained raters' impressions of rapport in each slice. The three slices were strongly correlated with each other, ranging from 0.60–0.82, suggesting good interchangeability. It was not stated, however, whether the slices were rated consecutively (one after the other). If that was the case, there could be some inflation due to carryover of a rater's impression from one slice to the next.

Murphy et al. (2015) investigated inter-slice reliability based on data from four separate studies in which specific nonverbal behaviors were coded in 30-s or 1-min slices from video recorded interactions that originally ranged from 5 to 9 min in length (though not all studies coded all behaviors). Inter-slice reliability (as assessed with intraclass correlations) was strongest for gazing behavior but slices of gestures, nods, self-touch, and smiles also reached reasonable levels of inter-slice reliability, providing empirical evidence of slice interchangeably for those measured behaviors. However, speaking time was a notable exception in showing considerable variation across studies and there was no evidence that one slice of speaking time reasonably predicted any other speaking time slice within an interaction.

As mentioned, with higher inter-slice reliability, a researcher can be more confident that thin slices are appropriate. For planning stages, researchers could consider applying the Spearman–Brown formula to calculate how many slices are needed to achieve a given reliability level for the slices combined (based on estimates from pilot data or past studies) (Brown, 1910; Spearman, 1910). The formula could also be applied *post-hoc*, essentially examining intercorrelations to see if they correlate well enough to justify combining them into a “total” (This is conceptually analogous to calculating reliability with Cronbach's α.). For further information on using the Spearman-Brown formula to establish inter-slice reliability see Li et al. (1996) and Murphy et al. (2015).

In general, there is relatively little comparative research specifically investigating inter-slice reliability. While the above research provides some evidence of slice interchangeability, such research did not answer questions about slice-length comparisons (e.g., 20 vs. 40 s slices, or 1 vs. 3 min, slices, etc.) or other measured behaviors or macro constructs. What is quite clear is that future research is needed to investigate inter-slice reliability and the related questions.

#### Slice-Whole Validity

Slice-whole validity is another way of examining representativeness, not between slices but between any given slice and the totality of the measured behavior. In comparison to inter-slice reliability research, there is a larger set of studies investigating slice-whole validity. Murphy (2005) investigated the slice-whole validity of five specific nonverbal behaviors (gestures, nods, self-touches, smiles, time spent gazing at partner) by coding 50 participants who engaged in 15-min dyadic social interactions. Reliable judges coded three randomly selected 1-min slices. A separate set of judges coded the full 15-min interactions for each behavior. With the exception of self-touch, 1-min slices showed acceptable slice-whole validity (i.e., moderate to large effects) based on part-whole correlations (which removes possible inflation from including a given slice from the behavior total). And all behaviors reached acceptable slice-whole validity when adding two of the randomly selected slices together. (This also true for summing three slices except for nodding behavior, which showed a consistent *decrease* in representativeness as slices were added together). In a more extensive analysis of four studies, Murphy et al. (2015) (described in previous section) found modest to strong slice-whole validity for gaze, nods, and smiles; yet, there was little evidence of such validity in speaking time and gestures (see Murphy et al., 2015 Figure 2). The location of the extracted slice mattered; generally, slices extracted from the middle of interactions showed stronger validity than slices from the beginning or end of an interaction.

Evidence of slice-whole validity also was found in several studies involving clinical settings. In Perrault (2020), participants viewed a clinician video biography and rated the clinician on various constructs such as trust and liking. Results showed that 46-s slices were equally predictive of ratings across eight constructs when compared to 63 and 80-s slices (which constituted the “whole” interaction). A corpus of videotaped patient-counselor clinical sessions (20–30 min) had previously been coded for verbal constructs such as open-ended questions and affirmation (Carcone et al., 2015). Each session was divided into four equal segments and then 1- or 2-min slices were randomly selected from each segment. There was considerable variation depending on the construct but the authors concluded that using six 2-min slices from across the longer interaction sufficiently captured the whole interaction. And slice-whole validity dropped only slightly when using four 2-min slices (compared to six 2-min slices). In a similar study, acceptable levels of slice-whole validity was found for 5-min, and especially 10-min, slices extracted from motivational clinical interviews (a type of trained counseling style) in five of seven measured constructs (Klonek et al., 2015). And, importantly, the authors also investigated the location of extracted slices and found that 5-min slices extracted from after the first 5-min of the interaction had the strongest validity, supporting previous recommendations to avoid using slices from the very beginning of interactions (Murphy et al., 2015; Hirschmann et al., 2018).

In another study, across five measured constructs (liking, attention, coordination, trust, rapport), there was evidence of slice-whole validity for 1.5-min slices of patient-physician interactions, though the effects were modest in magnitude (Foster, 2015)^6^. Caperton et al. (2018) investigated the minimum slice length needed to capture the whole of therapist behavior during motivational interviewing sessions. Previously recorded sessions (average length = 28 min) had been coded for therapist utterances. The authors found using ~8 min reliably captured the whole session. In another study, 10-min slices extracted from 40+ min mother-child interactions showed strong evidence of slice-whole validity in measuring maternal sensitivity and maternal feedback (Hirschmann et al., 2018). Such findings provide further support to the notion that smaller excerpts from longer behavioral streams can adequately represent some behaviors across an interaction.

As a whole, research generally suggests that excerpts shorter than their respective totals have the potential to reliably capture coded behaviors or constructs. But of course, any conclusions from the aforementioned slice-whole validity findings are qualified by the many other variables at play, including the total length of the interaction, the type of interaction, and constructs being measured. And there is research refuting slice-whole validity. James et al. (2012) recorded interactions between mothers and their deaf children and found that 3-min slices of play behaviors were not representative of whole 18-min interactions. The authors concluded: “If we had used 3 min segments [slices] to code data then our conclusions would have differed considerably from the findings based on an entire play session” (p. 357), providing a cautionary message against thin-slice coding for certain behaviors in specific contexts and/or with targeted populations.

### Thin-Slice Predictive Validity

The second comparative thin-slice tradition concerns predictive validity: How do slices fare, compared to total, in predicting a different outcome variable (that is, a variable that is different from the behavior that is measured in the slice)? As a hypothetical example, consider a study of nervousness and smiling during 15-min video recorded dyadic interactions. Targets complete a self-report measure of nervousness and judges count target smiles in 1-min slices across the entire 15-min interaction, enabling the researcher to calculate 1-min slices of smiling and the entire 15-min of smiling. If the correlation between target nervousness and the 1-min smile coding is close to or the same as the correlation between target nervousness and the 15-min smile coding, it would suggest that little to no predictive validity is lost by using the 1-min coding. We use the term “predictive” loosely, meaning the variable does not have to be measured literally after the behavior occurred. We also refer to the predicted variables as “outcome variables,” without implying the “outcome” has a causal relationship to the behavior that is measured.

Ambady and Rosenthal (1992) conducted a meta-analysis of correlations between behaviors coded from excerpts and a wide array of outcome variables. The authors found no association between slice length (which varied from under 30 s to 5 min) and the strength of the predictive correlations. Although this study was groundbreaking, not only in introducing the term “thin slices” but in showing that thin slices can predict other variables, it was not optimal for testing the impact of slice length—because the analysis was necessarily a comparison between studies rather than within studies, meaning that both slice lengths and outcome variables were confounded with other study variables (sample characteristics, for example) and therefore made for an imprecise test of the slice-length question.

Ambady and Rosenthal (1993) began the tradition of comparing slice lengths *within* a study. In two studies of thin slices of teacher behavior predicting performance evaluations, they compared 2, 5, and 10-s excerpts and found that although the correlations for longer slices were stronger, the longer slices did not predict to the criterion variable of teacher effectiveness better than did the shorter slices at statistically-significant levels, indicating evidence of predictive validity.

In Roter et al. (2011), three 1-min slices of verbal behavior (selected from early, middle, and late, as well as combined) were as predictive of independent judgments of rapport between clinicians and patients as was coding of the full 15-min interaction; also the single 1-min slices were not much different than the 3-min combined slice. In a different analysis based on the same database, Hall et al. (2009; described above) obtained ratings of rapport from three 1-min video slices. The individual slices and their 3-min total were compared in terms of correlations with a wide range of other variables. In general there was some loss of predictive validity for the 1-min slice (which was the 1st min of the interaction) compared to all 3 min, with some variables showing a fairly strong loss of prediction. However, for a number of variables the loss was not great or even non-existent, such as coder ratings of interest, warmth, and respect, and analog patients' ratings of the clinician's competence, calmness, communication quality, and self-confidence, as well as patients' own satisfaction.

Tskhay et al. (2017) obtained ratings of charisma from 5, 15, and 30-s silent slices from a 1-min video. There was not much difference between the slices and the total for predicting independent ratings of leadership potential and several other variables including gender, eye contact, wearing glasses, and physical attractiveness.

In a study of job applicants, slices (<130 s in length) applicant audio cues (e.g., prosody, pauses, etc.) predicted observer-rated hireability impressions based on the whole interview (Nguyen and Gatica-Perez, 2015), indicating that shorter excerpts of a job interview could be predictive of interview outcomes. Additionally, the results indicated that no one slice was markedly more predictive than another, though the thin slices were always less predictive than full interview outcomes. Similar results were found in analyzing participants in audio-recorded game interactions, whereby temporal position of 1-min slices from the beginning, middle, or end showed that any slice was equally predictive of game performance (Lepri et al., 2009).

Murphy et al. (2019) and Wang et al. (2020) offered multivariable examinations of predictive validity by examining multiple behaviors and multiple outcome variables. Murphy et al. (2019) examined predictive validity in five studies for six nonverbal behaviors (nodding, smiling, gesturing, gazing, self-touch, and speaking time). While 1-min slices were somewhat worse in predicting a highly varied list of 33 outcome variables than the whole 5-min videos were, 2-min slices were nearly as predictive as the 5-min totals. Wang et al. (2020) collected self-rated, perceiver-rated, and objectively measured data within one study based on a 5-min interaction. One-min slices were rated for verbal and nonverbal behaviors via global impressions, using the same rater for all five slices and also using a different rater for each slice. For single slices, results indicated no clear pattern for optimal slice locations. In general, single slices had weaker predictive validity than the total (5 slices combined). However, slices of 2 or 3 min were, in general, equal to 5-min total in predictive validity. The magnitude of correlations was similar when same- vs. different-coder methodologies were compared.

As a whole, research on thin-slice predictive validity suggests that thin-slice measurements may adequately predict an outcome variable, in comparison to variable measurement of an entire interaction. But once again, the preceding findings are qualified by many existing contingencies such as different measured constructs, outcome variables, slice lengths, and total interaction lengths, among other considerations. Researchers using the same specific behaviors and/or variables as the aforementioned studies could examine specific findings for more details on how to conduct their own behavioral measurement.

### Accuracy-Length Validity

There is a longstanding tradition of using thin slices as stimuli in studies of person perception accuracy. The study of accurate person perception can be considered a special case of predictive validity because accuracy is defined as the match or correlation between a perceiver's judgment and the criterion (i.e., the “correct answer” on a test item). Depending on the cue modality and the construct being judged, tests of interpersonal judgment accuracy vary considerably in the length of their stimuli, but not a great deal is known about the impact of variations in slice length. Many studies of emotion recognition use photographs, exposed for varying amounts of time, while others use dynamic stimuli of <20 s; example tests are the Diagnostic Analysis of Nonverbal Accuracy–Adult Prosody (DANVA2-AP; Baum and Nowicki, 1998), the Geneva Emotion Recognition Test (GERT; Schlegel et al., 2014), the Multimodal Emotion Recognition Test, MERT, Bänziger et al., 2009), and the Profile of Nonverbal Sensitivity (PONS; Rosenthal et al., 1979).

In the accuracy field, researchers' interest in slice length is twofold. First, for psychometric reasons a test developer might compare accuracy resulting from different slice lengths in order to create a test that has an optimal difficulty level. If the slices are too short, perhaps judgment accuracy is impossible, while if the slices are too long, the test might be too easy. For example, the PONS test was originally piloted with 5-s audiovisual clips, but this was reduced to 2 s to reach a psychometrically optimal difficulty level. Similarly, Gesn and Ickes (1999) reported choosing 15-s clips from dyadic interactions as stimuli in their judgment study, based on a process of trial and error in the piloting phase.

Slice length also has theoretical, not just methodological, interest to accuracy researchers because they want to know how accuracy of judging some attribute, state, or trait of target others may be related to the length of exposure to targets. Researchers interested in the validity of first impressions are especially likely to ask this question (Ambady and Skowronski, 2008). We know that people draw conclusions about others automatically and very quickly (Todorov, 2017). In other words, people rely intuitively on thin slices. In one study, almost a third of hiring managers reported making a decision about an applicant's suitability within the first 5 min of an interview (Frieder et al., 2016). And for many kinds of judgments, impressions are formed based on stimuli far shorter than that. Thus arises the question of how much time is needed to form an accurate judgment. Ambady et al. (1999) found that accuracy of judging sexual orientation was significantly greater for 10-s video clips than for 1-s clips. Rule and Ambady (2008) found that above-chance accuracy at judging sexual orientation could be obtained even when photographs were displayed for 50 ms but not for 33 ms. Some types of nonverbal affective stimuli can be judged very accurately from extremely minimal exposure length. The Japanese and Caucasian Brief Affect Recognition Test (JACBART; Matsumoto et al., 2000), which is based on prototypical, posed, “basic” emotions, achieved high judgment accuracy with exposures as short as 1/5 s. In contrast, spontaneously produced nonverbal affective expressions can be quite hard to judge, even at considerably longer exposures (Gesn and Ickes, 1999; Hall and Schmid Mast, 2007).

In the domain of personality judgment, researchers often use longer stimuli than those used for emotion recognition. In examining length of exposure, Blackman and Funder (1998) found that video clips of 25–30 min produced significantly greater accuracy in judging personality than clips of 5–10 min. However, there was almost equal accuracy for 5–10 min clips compared to 15–20 min. Letzring et al. (2006) found that interacting with someone for 3 hr did not produce more accurate personality judgment than interacting with someone for 50 min. And Fowler et al. (2009) looked at slices of 5, 10, and 20 s in a study of accuracy of judging psychopathy in criminal offenders. For one criterion measure of psychopathy, the *shortest* slice length produced the highest accuracy.

Carney et al. (2007) examined Big Five judgment accuracy for slices of 5, 20, 45, 60, and 300 s duration and found that extraversion and conscientiousness showed significant linear trends indicating increased accuracy for longer slices, and agreeableness showed a marginally significant linear trend. However, accuracy for neuroticism and openness to experience did not show a linear trend for slice length. Importantly, accuracy was above chance even at 5 s of exposure for all of the traits except agreeableness. There was a gain in accuracy in going from 60 to 300 s, but it was not substantive. Similar results were found in a study comparing 30-s, 1-, 3-, and 5-min slices in accurately perceiving personality traits (Krzyzaniak et al., 2019). When analyzing all traits combined, accuracy (referred to as distinctive accuracy within the study) did not improve with longer slice length, but there was notable exceptions within specific traits, suggesting that appropriate slice lengths depend on the construct being measured.

Hall et al. (2008), in a meta-analysis on interpersonal accuracy studies, performed an analysis of slice length across studies. Exposure lengths ranging from <1 s to 45 min (including studies using photographs) revealed no evident trend associating slice length to judgment accuracy. However, the studies varied widely in terms of what construct was being judged (emotion, traits, etc.), and between-studies comparisons of slice lengths are confounded by all of the other methodological differences between the studies, potentially obscuring the duration effect.

Only one study that we know of has looked at accuracy and slice length for textual material. Hall et al. (2021) divided college students' two-page personal narratives into fifths and looked at individual fifths (slices) as well as cumulative slices in terms of readers' accuracy of judging the Big Five traits. For extraversion, agreeableness, and openness to experience, longer cumulative slices produced more accurate judgment, but this was not consistently the case: for neuroticism all of the cumulative slices showed higher accuracy than the total narrative did, due to the fifth and final slice producing no accuracy at all.

An unexplored question is how the criterion used for judging what is the “correct answer” on such a test might affect accuracy overall, and, relevant to our current interests, accuracy for different slice lengths or locations. The criterion for studies of judging personality is typically the target's self-report of personality, sometimes supplemented with reports by friends or family. For judging attributes of people there are often objective criteria that can be used; for example, if hierarchical status is being judged, the researcher might have access to the organizational chart of the company in question. For judging affective states, a wide variety of criteria are used including giving the target an assigned emotion to portray either by posing or by re-enactment of a lived experience, manipulation of situational stimuli such as what kind of photo or movie the target is watching, and the consensus of viewers (including the researchers) as to what affective state is being shown.

## (Many) Unanswered Questions

Like automated methods, the thin-slice method is not a panacea to solving coding burdens. It is impossible to state that thin slices would work for all behaviors. In fact, the research is quite clear that there is considerable variability depending on a number of factors. The measured construct may not be reliably measured via thin slices, which may be due to the construct's consistency or frequency of expression (Leikas et al., 2012; Murphy et al., 2015). Unique settings and/or specialized populations may not allow for adequate capture of behaviors via thin slices, as shown by the lack of slice-whole validity in play behaviors of deaf children with their mothers (James et al., 2012). Alternative measurements of the same construct (e.g., counting vs. rating, or intervals vs. whole) might not always work equally well or show parity within thin-slice measurement (Blanch-Hartigan et al., 2018).

Conclusions about appropriate slice length and location of the slice also cannot be universally applied. One might predict that longer slices could yield higher levels of representativeness, predictive validity, and accuracy. Unless a behavior is manifested with extreme reliability over time, there is inevitably an information loss when using shorter excerpts to represent a longer interaction. However, research also shows that the magnitude of that loss could be negligible, depending on the measured construct and slice length (Murphy, 2005; Carcone et al., 2015; Murphy et al., 2015). Research on behavioral consistency also reiterates that aggregation of data (e.g., behavioral measurements) from across situations, targets, and judges or coders increases the reliability of findings (Epstein, 1979, Moskowitz and Schwarz, 1982). While some predictive validity research suggests that the temporal position of the slice may not matter (Lepri et al., 2009; Nguyen and Gatica-Perez, 2015), selecting slices from after the beginning of an interaction (e.g., after the 1st min) has some empirical support, as there is some literature indicating lower slice-whole validity from very beginning slices (Klonek et al., 2015; Murphy et al., 2015).

The source of behavioral streams is an area worth further investigation. Much of the research cited here involved thin slices extracted from video stimuli recorded in a laboratory. Yet, thin-slice work is relevant beyond laboratory interactions. Thin-slice research exists across a wide array of domains such as judgments of online social networks, televised soccer and sport matches, TED talks, teachers' classroom behavior, and prison interviews, among many content areas (Fowler et al., 2009; Pretsch et al., 2013; Furley and Schweizer, 2014; Stopfer et al., 2014; Gheorghiu et al., 2020). As more evidence accumulates for the reliability and validity of thin-slice methods, it will be important that future comparative thin-slice research investigate stimuli from beyond the laboratory.

The vast majority of thin-slice research, whether comparative in nature or otherwise, involves White individuals from European and/or American backgrounds. And almost all of that research is limited to young adults or children. There are cross-cultural comparisons of person perception processes using thin slices. Using 10-s slices extracted from 3-min interactions, Place et al. (2012) found consistent levels of accuracy in detecting speed daters' romantic interest in samples from the U.S., Germany, and China. Thin-slice research showed equivalent levels of accuracy in judging rapport in U.S. and Greek participants (Bernieri and Gillis, 1995) and consensus in personality impressions were found in both U.S. and Chinese participant samples (Albright et al., 1997). Such studies at least extend thin-slice work into broader population samples, but the numbers are few and far between. And such research does little to acknowledge racial and/or ethnic identities even within the measured samples (Roberts et al., 2020). We are not aware of any comparative thin-slice research involving participants who are not predominantly White, European, and/or American. It is quite clear that there are likely cultural factors of targets or even coders that could limit any generalizability into populations not previously studied (Masuda et al., 2020).

## Conclusions

The thin-slice measurement technique itself is applicable to any behavioral domain, potentially even for behavioral measurement of non-human populations (Jamieson et al., 2017). Perhaps an expanded view of what constitutes a “thin slice” (beyond 5 min) is warranted given research on longer slices (e.g., 10 min) from lengthier interactions (e.g., >40 min) that shows similarities to findings examining shorter slices from briefer interactions (e.g., 30 s from 5 min) (Caperton et al., 2018; Hirschmann et al., 2018). At a conceptual level, there is evidence that thin slices reliably and validly measure behavior across various domains, including zero-acquaintanceship interactions and clinical settings.

At this initial stage, comparative thin-slice research provides some cautious optimism for researchers concerned with slice-whole validity and predictive validity, and those who use thin slices in interpersonal accuracy research. Appendix A is a representative list of cited studies on comparative thin-slice research. The Appendix is provided as potential resource for other thin-slice researchers who seek further information about reliability and validity of thin-slice measurement decisions. (It is important to note that the list is not intended to be exhaustive and the listed studies do not necessarily indicate support for the comparative construct). We also suggest reviewing the previously-mentioned factors listed in the “Deciding to Use Thin Slices for Behavioral Measurement” section and using the Spearman-Brown formula as discussed in the “Representativeness” section (see also Li et al., 1996; Murphy et al., 2015).

Given the current replication crisis in psychology (and beyond), the use of sound research practices is now more important than ever (Schimmack, 2020). Without reliable and valid measurement, any conclusions based on such measurements are acutely curtailed, if not nullified (Flake et al., 2017; Eronen and Bringmann, 2021). Of course, it is inaccurate to state that thin slices can be used any time a researcher wishes to reduce coding burdens by coding shorter excerpts of behavior. And, every researcher needs to make their own decision about whether a given degree of representativeness, predictive validity, or accuracy-length validity is “good enough” for their research purposes. Measurement is never perfect; each researcher decides at what point their measurements satisfy their standards and their resources. We hope this article may be a potential resource for researchers considering using thin-slice behavioral measurement; by reviewing current comparative thin-slice literature, researchers could identify potential sources which may support the many decisions going into using thin slices to measure behavior.

## Author Contributions

All authors contributed equally to the writing the manuscript and approved the submitted manuscript.

## Conflict of Interest

The authors declare that the research was conducted in the absence of any commercial or financial relationships that could be construed as a potential conflict of interest.

## Footnotes

^1^Although photos have been extensively used in person perception and impression formation research, there is a dearth of comparative thin-slice research involving photos—such as comparing photos to dynamic stimuli, or comparing longer or shorter exposures to photos. Thus, this paper focuses exclusively on dynamic stimuli.

^2^One literature we do not address concerns the “acquaintance effect” (Connelly and Ones, 2010), where accuracy of personality judgment is compared between people who know the target for longer or shorter amounts of time.

^3^Ambady and Rosenthal (1992) referred to such correlations as reflecting the accuracy of thin-slice judgments, but in fact such correlations speak to predictive validity.

^4^Retrieved February 2, 2021 per Google Scholar.

^5^It is likely that many researchers compare a variety of different slice lengths or locations in the process of developing their methodology but do not report that entire process in their publications. Therefore, there may be informal comparative thin-slice research in existence that we are not aware of.

^6^Foster (2015) concluded that thin-slice ratings were *not* comparable to full interaction ratings. However, the magnitude of their effects (*r*s between 0.33 and 0.49) align with other comparative thin-slice research findings on slice-whole validity.
