# Supplementary material for: Capturing Behavior in Small Doses: A Review of Comparative Research in Evaluating Thin Slices for Behavioral Measurement
Source: Front Psychol. 2021 Apr 29;12:667326. doi: 10.3389/fpsyg.2021.667326 (PMC8116694; doi:10.3389/fpsyg.2021.667326)
Supplement: Supplementary file 1 [file Table_1.pdf]

# Appendix

***Representative Comparative Thin-Slice Research Studies***

This table is provided as a resource to locate relevant studies regarding the reliability and validity of thin slices and is not intended to be exhaustive of all comparative thin-slice research. Importantly, the final column simply indicates whether the construct was examined and does *not* indicate that the study necessarily found support for the comparative construct. In many cases, a study found support for some measured variables or constructs but not others. We encourage researchers to review specific studies if looking for evidence or justification of their own slice decisions.

| Article | Description | Slice length(s) | Variable(s) | Comparative construct |
|---|---|---|---|---|
| Ambady et al. (1999) | Student discussions of campus activities | 1 and 10 s | Sexual orientation | Accuracy-length validity |
| Ambady & Rosenthal (1993) | High-school and college teachers' class lectures | 2, 5, and 10 s | 15 molar behaviors with teacher effectiveness | Predictive validity |
| Blackman & Funder (1998) | Friend and zero-acquaintanceship dyadic interactions | 5 to 25 min | Personality profiles | Accuracy-length validity |
| Caperton et al. (2018) | Client-therapist counseling sessions | ~8 min | Therapist MI codes for: reflection-to-question ratio, complex reflections, open questions, MI-consistent responses | Slice-whole validity |
| Carcone et al. (2015) | Patient-counselor clinical interactions | 1 or 2 min | 20 MI patient and counselor communication codes | Slice-whole validity |
| Carney et al. (2007) | Zero-acquaintanceship interactions | 5, 20, 45, 60 s | Personality traits and affect | Accuracy-length validity |
| Foster (2015) | Patient-physician interactions | 1.5 min | Attention, coordination, liking, rapport, trust | Slice-whole validity |
| Fowler et al. (2009) | Prison inmate interviews | 5, 10, and 20 s | Psychopathy | Accuracy-length validity |
| Hall et al. (2008) | Database of 109 studies | <1 s to 45 min | Various states and traits | Accuracy-length validity |
| Hall et al. (2009) | Patient-medical student interactions | 1 and 3 min | Rapport | Inter-slice reliability; predictive validity |

| | | | | |
|---|---|---|---|---|
| Hall et al. (2014) | Technical support calls | 2 min | Satisfaction | Inter-slice reliability |
| Hall et al. (2021) | Written personal narratives | 281 to 1299 words | Big 5 personality traits | Accuracy-length validity |
| Hirschmann et al. (2018) | Mother-child interactions | 10 min | Maternal feedback, joint attention, sensitivity | Slice-whole validity |
| James et al. (2012)[a] | Mother-deaf child interactions | 3 min | Gaze, vocalizations | Slice-whole validity |
| Klonek et al. (2015) | Clinical interviews | 5 and 10 min | 7 MI verbal codes | Slice-whole validity |
| Krzyzaniak et al. (2019) | Zero-acquaintanceship group interactions | 30 s; 1, 3, and 5 min | Big Five personality traits | Accuracy-length validity |
| Lepri et al. (2009) | Group game interactions | 1 min | Speaker emphasis, activity, influence, mimicry, body/hand movement with game performance | Predictive validity |
| Letzring et al. (2006) | Zero-acquaintanceship group interactions | 50 min | Personality profiles | Accuracy-length validity |
| Murphy (2005) | Zero-acquaintanceship dyadic interactions | 1 min | Gaze, gestures, nods, self-touch, smiles | Slice-whole validity |
| Murphy et al. (2015) | 4 studies; various zero-acquaintanceship dyadic interactions | 30 s to 4.5 min | Gaze, gestures, nods, self-touch, smiles, speaking time | Inter-slice reliability; slice-whole validity |
| Murphy et al. (2019) | 5 studies; various zero-acquaintanceship dyadic interactions | 1, 2, 3, and 4 min | Gaze, gestures, nods, self-touch, smiles, speaking time with 33 various outcome variables | Predictive validity |
| Nguyen & Gatica-Perez (2015) | Job interviews | 2 s to 2 min | Speaking behaviors, body and head movements with hireability impressions | Predictive validity |
| Perrault (2020) | Clinician biography videos | 46 and 63 s | Patient ratings of anticipated satisfaction, behavioral intention, ease of selection, expertise, likeability, trust, uncertainty | Slice-whole validity |
| Roter et al. (2011) | Patient-medical student interactions | 1 and 3 min | Various rapport-based and verbal behaviors | Slice-whole validity; predictive validity |

| | | | | |
|---|---|---|---|---|
| Satterstrom et al. (2019) | Student groups in decision-making tasks | 10, 30, and 60 s | Group effectiveness | Accuracy-length validity |
| Tskhay et al. (2017) | Persuasive speeches | 5, 15, and 30 s | Leadership variables, speaker appearance variables, with speaker charisma | Predictive validity |
| Wang et al. (2019) | Zero-acquaintanceship dyads | 1, 2, 3, and 4 min | Smiling, nodding, leaning in, humor/telling stories, speaking about self with various outcome variables | Predictive validity |

*Note*. Accuracy-length validity = whether interpersonal judgment accuracy depends on the length of the slice. Predictive validity = how well thin slices can predict variables that are different from the behavior measured in the slice. MI = Motivational Interviewing counseling style (Levounis et al., 2017). Slice-whole validity = whether thin slices can adequately approximate the total of the recorded behavior. Inter-slice reliability = how interchangeable individual slices are in relation to one another.

[a] See James et al. (2012) Table 1 for a list of studies using thin slices in parent–child interactions.

**Appendix References**

Ambady, N., & Rosenthal, R. (1992). Thin slices of expressive behavior as predictors of interpersonal consequences: A meta-analysis. *Psychological Bulletin*, *111*(2), 256–274. https://doi.org/10.1037/0033-2909.111.2.256

Ambady, N., & Rosenthal, R. (1993). Half a minute: Predicting teacher evaluations from thin slices of nonverbal behavior and physical attractiveness. *Journal of Personality and Social Psychology*, *64*(3), 431–441. https://doi.org /10.1037/0022-3514.64.3.431

Blackman, M. C., & Funder, D. C. (1998). The effect of information on consensus and accuracy in personality judgment. *Journal of Experimental Social Psychology, 34*(2), 164–181. https://doi.org/10.1006/jesp.1997.1347

Caperton, D. D., Atkins, D. C., & Imel, Z. E. (2018). Rating motivational interviewing fidelity from thin slices. *Psychology of Addictive Behaviors, 32*(4), 434–441. https://doi.org/10.1037/adb0000359

Carcone, A. I., Naar, S., Eggly, S., Foster, T., Albrecht, T. L., & Brogan, K. E. (2015). Comparing thin slices of verbal communication behavior of varying number and duration. *Patient Education and Counseling*, *98*(2), 150–155. https://doi.org/10.1016/j.pec.2014.09.008

Carney, D. R., Colvin, C. R., & Hall, J. A. (2007). A thin slice perspective on the accuracy of first impressions. *Journal of Research in Personality, 41*(5), 1054–1072. https://doi.org/10.1016/j.jrp.2007.01.004

Foster, T. S. (2015). The reliability and validity of the thin slice technique: Observational research on video recorded medical interactions [ProQuest Information & Learning]. In *Dissertation Abstracts International: Section B: The Sciences and Engineering* (Vol. 75, Issue 8–B(E)).

Fowler, K. A., Lilienfeld, S. O., & Patrick, C. J. (2009). Detecting psychopathy from thin slices of behavior. *Psychological Assessment*, *21*(1), 68–78. https://doi.org/10.1037/a0014938.supp (Supplemental)

Hall, J. A., Andrzejewski, S. A., Murphy, N. A., Mast, M. S., & Feinstein, B. A. (2008). Accuracy of judging others' traits and states: Comparing mean levels across tests. *Journal of Research in Personality*, *42*(6), 1476–1489. https://doi.org/10.1016/j.jrp.2008.06.013

Hall, J. A., Harvey, S. E., Johnson, K. E., & Colvin, C. R. (2021). Thin-slice accuracy for judging Big Five traits from personal narratives. *Personality and Individual Differences, 171*, 110392. https://doi.org/10.1016/j.paid.2020.110392

Hall, J. A., Roter, D. L., Blanch, D. C., & Frankel, R. M. (2009). Observer-rated rapport in interactions between medical students and standardized patients. *Patient Education and Counseling, 76*(3), 323-327. https://doi.org/10.1016/j.pec.2009.05.009

Hall, J. A., Verghis, P., Stockton, W., & Goh, J. X. (2014). It takes just 120 seconds: Predicting satisfaction in technical support calls. *Psychology & Marketing, 31*(7), 500–508. https://doi.org/10.1002/mar.20711

Hirschmann, N., Kastner-Koller, U., Deimann, P., Schmelzer, M., & Pietschnig, J. (2018). Reliable and valid coding of thin slices of video footage: Applicability to the assessment of mother-child interactions. *Journal of Psychopathology and Behavioral Assessment, 40*, 249-258. https://doi.org/10.1007/s10862-017-9630-x

James, D. M., Wadnerkar, M. B., Lam-Cassettari, C., Kang, S., & Telling, A. L. (2012). Thin slice sampling of video footage for mother/child interaction: Application to single

cases. *Journal of Psychopathology and Behavioral Assessment*, *34*(3), 351–360. https://doi.org/10.1007/s10862-012-9282-9

Klonek, F. E., Quera, V., & Kauffeld, S. (2015). Coding interactions in Motivational Interviewing with computer-software: What are the advantages for process researchers? *Computers in Human Behavior, 44,* 284-292. https://doi.org/10.1016/j.chb.2014.10.034

Krzyzaniak, S. L., Colman, D. E., Letzring, T. D., McDonald, J. S., & Biesanz, J. C. (2019). The effect of information quantity on distinctive accuracy and normativity of personality trait judgments. *European Journal of Personality, 33*(2), 197-213. https://doi.org/10.1002/per.2196.

Lepri, B., Mana, N., Cappelletti, A., & Pianesi, F. (2009). Automatic prediction of individual performance from "thin slices" of social behavior. In *Proceedings of the 17th ACM International Conference on Multimedia*, 733–736. https://doi.org/10.1145/1631272.1631400

Letzring, T. D., Wells, S. M., & Funder, D. C. (2006). Information quantity and quality affect the realistic accuracy of personality judgment. *Journal of Personality and Social Psychology*, *91*(1), 111–123. https://doi.org/10.1037/0022-3514.91.1.111

Levounis, P., Arnaout, B., & Marienfeld, C. (2017). *Motivational interviewing for clinical practice* (P. Levounis, B. Arnaout, & C. Marienfeld (Eds.)). American Psychiatric Publishing, Inc. https://doi.org/10.1176/appi.books.9781615371860

Murphy, N. A. (2005). Using thin slices for behavioral coding. *Journal of Nonverbal Behavior, 29*(4), 235-246. https://doi.org/10.1007/s10919-005-7722-x

Murphy, N. A., Hall, J. A., Mast, M. S., Ruben, M. A., Frauendorfer, D., Blanch-Hartigan, D., Roter, D. L., & Nguyen, L. (2015). Reliability and validity of nonverbal thin slices in social interactions. *Personality and Social Psychology Bulletin*, *41*(2), 199–213. https://doi.org/10.1177/0146167214559902

Murphy, N. A., Hall, J. A., Ruben, M. A., Frauendorfer, D., Schmid Mast, M., Johnson, K. E., & Nguyen, L. (2019). Predictive validity of thin-slice nonverbal behavior from social interactions. *Personality and Social Psychology Bulletin*, *45*(7), 983–993. https://doi.org/10.1177/0146167218802834

Nguyen, L. S., & Gatica-Perez, D. (2015). I would hire you in a minute: Thin slices of nonverbal behavior in job interviews. In *Proceedings of the 2015 ACM on International Conference on Multimodal Interaction (ICMI '15), Association for Computing Machinery* (pp. 51-58). https://doi.org/10.1145/2818346.2820760

Perrault, E. K. (2020). The diminishing returns for longer healthcare provider video biographies: A thin slice examination of patient decision-making. *Health Communication*, *36*(5), 650-658. https://doi.org/10.1080/10410236.2020.1733230

Roter, D. L., Hall, J. A., Blanch-Hartigan, D., Larson, S., & Frankel, R. M. (2011). Slicing it thin: New methods for brief sampling analysis using RIAS-coded medical dialogue. *Patient Education and Counseling*, *82*(3), 410–419. https://doi.org/10.1016/j.pec.2010.11.019

Satterstrom, P., Polzer, J. T., Kwan, L. B., Hauser, O. P., Wiruchnipawan, W., & Burke, M. (2019). Thin slices of workgroups. *Organizational Behavior and Human Decision Processes*, *151*, 104–117. https://doi.org/10.1016/j.obhdp.2018.12.007

Tskhay, K. O., Zhu, R., & Rule, N. O. (2017). Perceptions of charisma from thin slices of behavior predict leadership prototypicality judgments. *The Leadership Quarterly*, *28*(4), 555–562. https://doi.org/10.1016/j.leaqua.2017.03.003

Wang, M. Z., Chen, K., & Hall, J. A. (2020). Predictive validity of thin slices of verbal and nonverbal behaviors: Comparison of slice lengths, locations, and rating methodologies. *Journal of Nonverbal Behavior, 44*, 53-66. https://doi.org/10.1007/s10919-020-00343-1
